# Magnetic Resonance Imaging of Orbital Solitary Fibrous Tumors: Radiological-Pathological Correlation Analysis

**DOI:** 10.5334/jbsr.2097

**Published:** 2021-03-16

**Authors:** Ryuhei Masuno, Daisuke Yunaiyama, Yukiko Shishido-Hara, Daisuke Yoshimaru, Chifumi Maruyama, Yoichi Araki, Hiroshi Goto, Toshitaka Nagao, Kazuhiro Saito

**Affiliations:** 1 Tokyo Medical University, JP

**Keywords:** solitary fibrous tumor, MRI, texture analysis, orbit

## Abstract

**Background:**

Solitary fibrous tumors (SFTs) are rare and can be misdiagnosed because of their various radiological appearances.

**Purpose:**

To clarify the characteristic MRI findings of SFTs by analyzing their radiological-pathological correlation.

**Material and Methods:**

Nine consecutive patients with SFT who underwent magnetic resonance imaging (MRI) prior to surgery were analyzed. Eight patients underwent contrast-enhanced MRI, and three underwent dynamic MRI. Radiological-pathological correlation analysis, co-occurrence matrix, run-length matrix, and histogram analysis were performed to assess the relationship between pathological findings T1- and T2-weighted images (T1-WI and T2-WI).

**Results:**

All nine lesions ranged in size from 20 to 36 mm. Seven lesions were located in the superior portion of the retrobulbar space found outside of the muscle cone, and two lesions in the inferior portion were located within it. No significant correlation was observed between the amount of collagenous tissue and the qualitative evaluation of the signal on T1-WI and T2-WI. Kurtosis on T2-WI was significantly correlated with the amount of collagenous tissue (*ρ* = –0.97, *p* < 0.0001) and endothelial cells (*ρ* = –0.49, *p* = 0.0479).

**Conclusion:**

Kurtosis in the histogram analysis on T2WI showed a strong correlation with the amount of collagenous tissue.

## Introduction

Solitary fibrous tumors (SFTs) are rare spindle-cell neoplasms originating from the mesenchymal tissue. SFTs were initially thought to occur exclusively in the intrathoracic region; however, they have recently been reported to occur in extra-pleural sites, including the orbit [1]. Although once grouped with hemangiopericytomas, SFTs have been reclassified as a separate category of tumors [2]. Orbital SFTs were first identified in 1994. Their histopathology has subsequently been described in detail [3]. Local recurrence and malignant transformation occur more frequently in orbital SFTs than in orbital schwannomas [4,5]. Therefore, complete excision and long-term follow-up are necessary. Zhang et al. reported that the marked enhancement displayed in hyperintense lesions and the isointense areas displayed on T2-WI with washout are characteristics that may help differentiate SFTs from other tumors in the orbit [6]. However, there are few reports describing the association between imaging data and the pathology of SFTs. Recently, texture analysis has been used to detect features of images that are difficult to determine by visual observation. Some reports have shown detailed tissue characterization using texture analysis for radiological image [7]. Therefore, the purpose of this study was to clarify the characteristic MRI findings of orbital SFTs by conducting a radiological-pathological correlation analysis.

## Materials and methods

This retrospective study was conducted in accordance with the World Medical Association Declaration of Helsinki and was approved by the institutional review board. Informed consent was waived.

### Subjects

The data of subjects were collected from a pathology database, using the key words “orbit” and “solitary fibrous tumor” within the previous 10 years. All SFTs were resected and confirmed pathologically. Extra orbital tumors were excluded from the study. Among the patients with SFTs, MRI was performed on nine patients, who were subsequently included in this study. The nine patients comprised three men and six women, ranging in age from 14 to 77 years (median: 49 years).

### MRI parameters

MRI was performed with a 1.5-Tesla (T) scanner (Avanto, Siemens, Erlangen, Germany). The MRI analysis included T1-weighted (T1-WI), T2-weighted (T2-WI), and diffusion-weighted imaging. Contrast-enhanced MRI was performed in 8 patients, and dynamic MRI was performed in three patients. T1-WI was performed under the following parameters: Repetition time (TR): 450 msec, Echo time (TE): 12 msec, Bandwidth (BW): 100 Hz, Flip angle (FA): 90°, matrix: 204 × 256 and Field of view (FOV): 150 × 150 m. A T2-WI with fat suppression was performed under the following parameters: TR: 3500 msec, TE: 96 msec, BW: 65 Hz, FA: 170°, matrix: 234 × 320 and FOV: 150 × 150 mm. A diffusion-weighted image was performed under the following parameters: TR: 4500 msec, TE: 71 msec, BW: 100 Hz, FA: 180°, matrix: 166 × 166 and FOV: 280 × 280 mm. A contrast-enhanced T1-WI was performed under the following parameters: TR: 650 msec, TE: 12 msec, BW: 120 Hz, FA: 90°, matrix: 204 × 256 and FOV: 150 × 150 mm.

### Image analysis

Two experienced radiologists (with 8 and 28 years of experience) performed the consensus readings. The following characteristics were evaluated from the MRI: Contour, location, and signal intensity on T1-WI or T2-WI, linear or curvilinear hypointense line in the lesion, and enhancement pattern. Signal intensities were determined by comparing them with the signal intensity of the cerebral cortex. Contours were classified as either well-defined or irregular. Location was classified as either above or below the center, and as either inside or outside of the muscle cone. Signal intensities on T1-WI and T2-WI were classified into 5 categories. Hypointensity was categorized as 1, isointensity was categorized as 3, and hyperintensity was categorized as 5. Category 2 was defined as samples showing a combination of hypointensity and isointensity. Category 4 was defined as samples showing mixed isointensity and hyperintensity. The curvilinear hypointense line was evaluated and classified as either present or absent. The enhancement pattern in the equilibrium phase was classified as either homogeneous or heterogeneous enhancement.

Radiologists and radiological technologists delineated the region of interest on T1-WI and T2-WI. The ROIs were set at the entire tumor through all slices on T1-WI and T2-WI. The data acquired from each slice were summated to derive pixel-by-pixel images. Three different texture features (histogram, co-occurrence matrix, and run-length matrix) were extracted on a program developed using MATLAB after saving DICOM data into local storage.

Pathological evaluations were performed by an experienced pathologist (more than 20 years of experience). Azan and Masson trichrome staining methods were used to observe the proliferation of collagenous fibers or fibrous components [8] and the subjects were classified into 4 categories as follows: (1) myxoid change, (2) small amount of collagenous tissue, (3) moderate amount of collagenous tissue and (4) abundant collagenous tissue. Immunohistochemical staining of CD31 was performed to evaluate vascular richness. The positive rates of CD31 were classified into the following 3 categories: 1 poor, 2 moderate, and 3 rich.

Two radiologists and one pathologist discussed the findings and performed radiological-pathological correlation analyses between areas of interest.

### Statistical analysis

Data are expressed as mean ± standard deviation. The radiological-pathological correlation was estimated. Spearman’s rank correlation coefficient was performed to compare MRI findings and texture analysis with pathological findings. A *p*-value of less than 0.05 was considered to indicate a statistically significant difference.

## Results

All nine lesions ranged in size from 20 to 36 mm (median: 28 mm).

### MRI findings

The classified categories of MRI findings are summarized in ***Table 1***. The contour of the lesion was well-defined in six lesions and irregular in three lesions. Regarding the location, seven lesions were found to be in the superior portion, and two lesions were in the inferior portion. Seven lesions were inside the muscle cone, and two lesions were outside the muscle cone. On T1-WI, homogeneous isointensity was displayed in four lesions, heterogeneous isointensity and hypointensity in two lesions, and heterogeneous isointensity and hyperintensity in three lesions. On T2-WI, marked homogeneous hyperintensity was displayed in one lesion, heterogeneous isointensity and hyperintensity in four lesions, homogeneous isointensity in one lesion, heterogeneous isointensity and hypointensity in two lesions and marked hypointensity in one lesion. A linear or curvilinear hypointense line was observed in six lesions and was absent in three lesions. A homogeneous enhancement pattern in the equilibrium phase was displayed in six lesions and a heterogeneous enhancement in two lesions.

**Table 1:** Detail classification of MRI findings.


MRI FINDINGS	CLASSIFICATION	NUMBERS

Contour	Well-defined	6

	Irregular	3

Location	Superior portion	7

	Inferior portion	2

	Inside muscle cone	2

	Outside muscle cone	7

Signal intensity on T1WI	Hypo intensity	0

	Hypo-Iso intensity	2

	Iso intensity	4

	Iso-Hyper intensity	3

	Hyper intensity	0

Signal intensity on T2WI	Hypo intensity	1

	Hypo-Iso intensity	2

	Iso intensity	1

	Iso-Hyper intensity	4

	Hyper intensity	1

Linear of curvilinear hypo-intensity line	Present	6

	Absent	3

Enhancement	Homogenous	6

	Heterogenous	2


T1WI (T1-weighted image), T2WI (T2-weighted image).

### Radiological-pathological correlation

**Figure 1:**
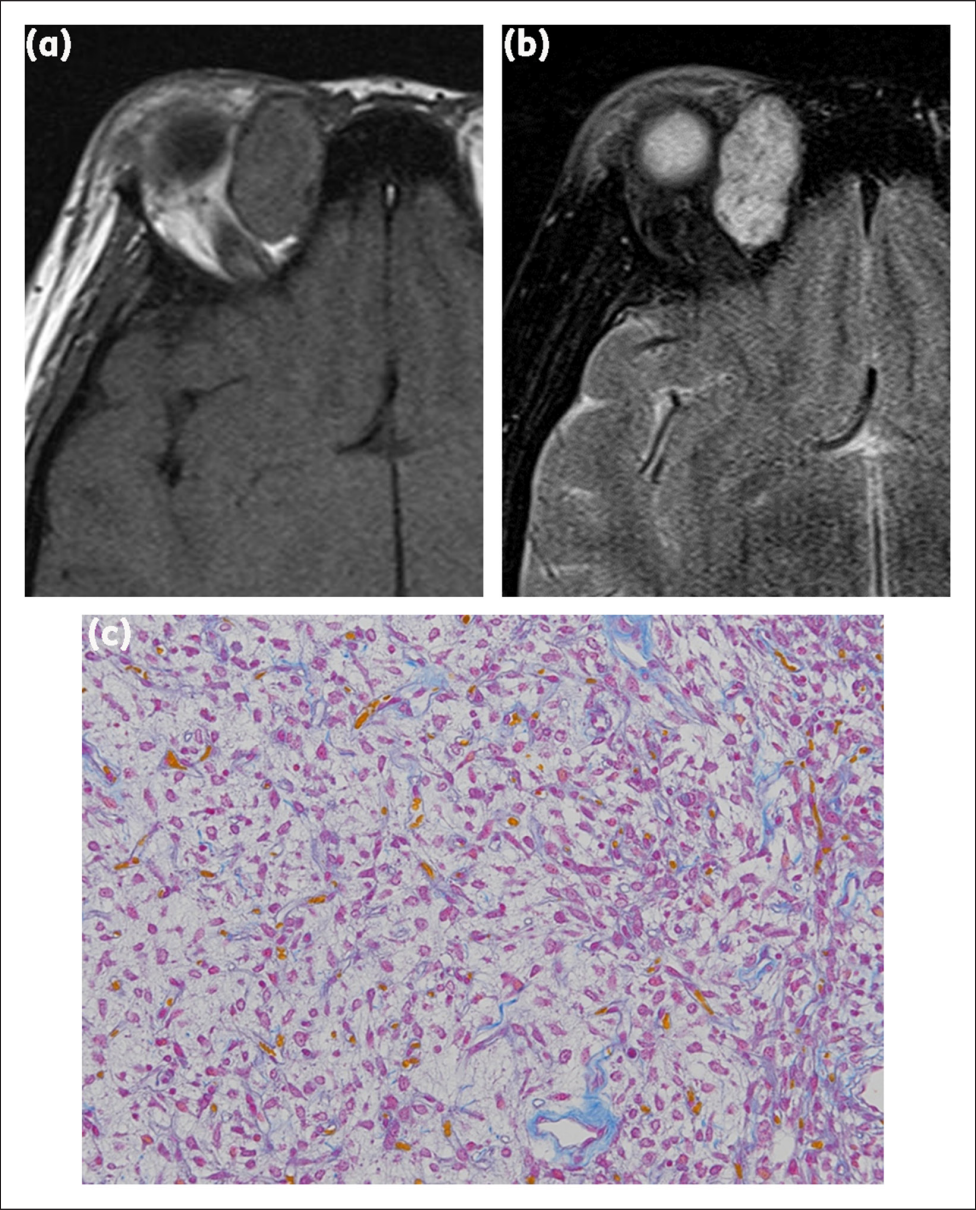
A 14-year-old woman with solitary fibrous tumor. **(a)** The tumor was located outside of the muscle cone and displayed isointensity compared with brain white mater on T1-weighted imaging. **(b)** The tumor displayed hyperintensity on T2-weighted imaging. **(c)** Hematoxylin and eosin staining of the tumor showing a prominent myxoid change.

**Figure 2:**
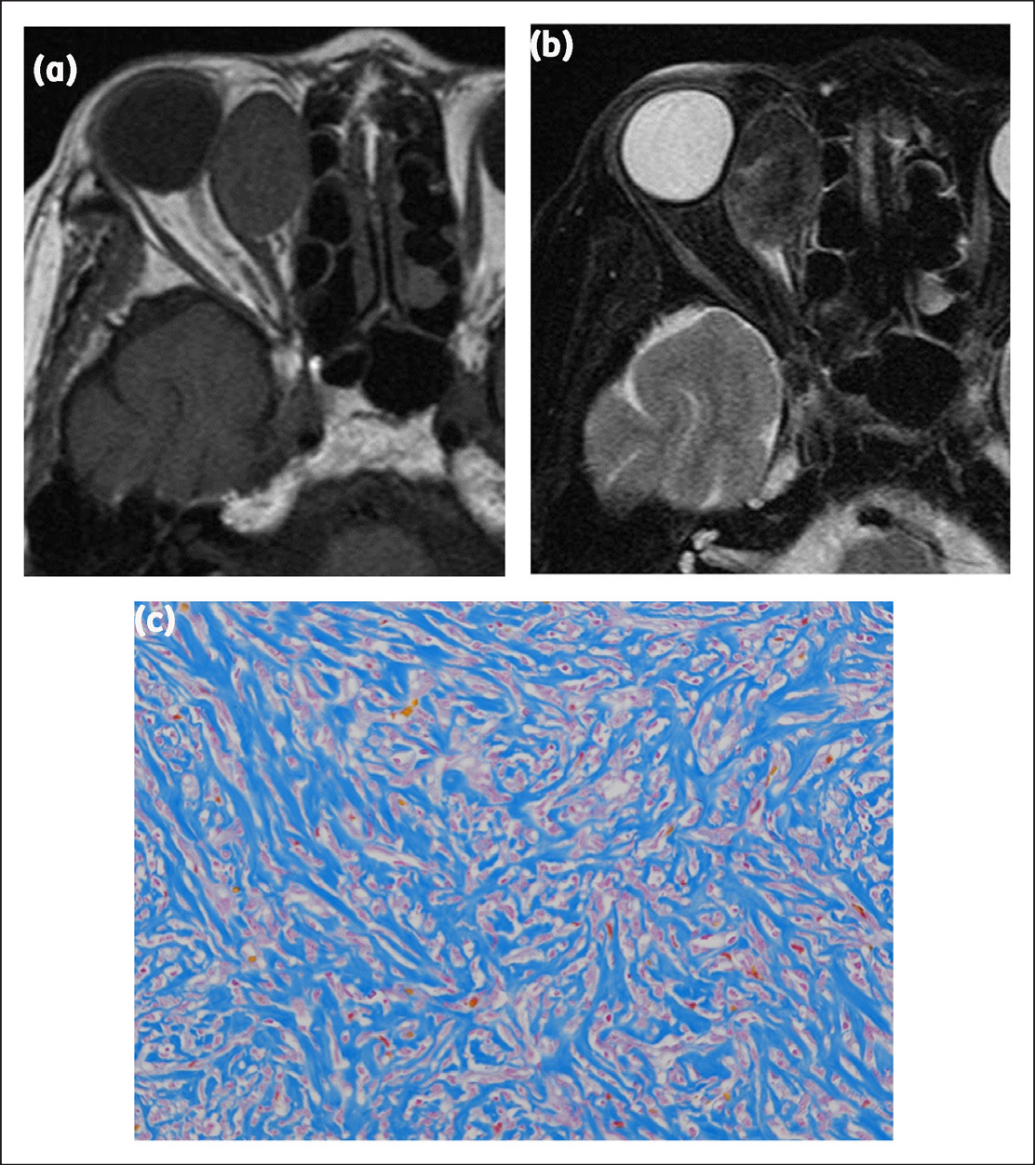
A 77-year-old woman with solitary fibrous tumor (SFT). **(a)** The tumor was located outside of the muscle cone and showed marked isointensity on T1-weighted imaging. **(b)** The tumor displayed hypointensity on T2-weighted imaging. **(c)** Azan staining of the tumor revealing a large amount of collagenous tissue in the lesion.

**Figure 3:**
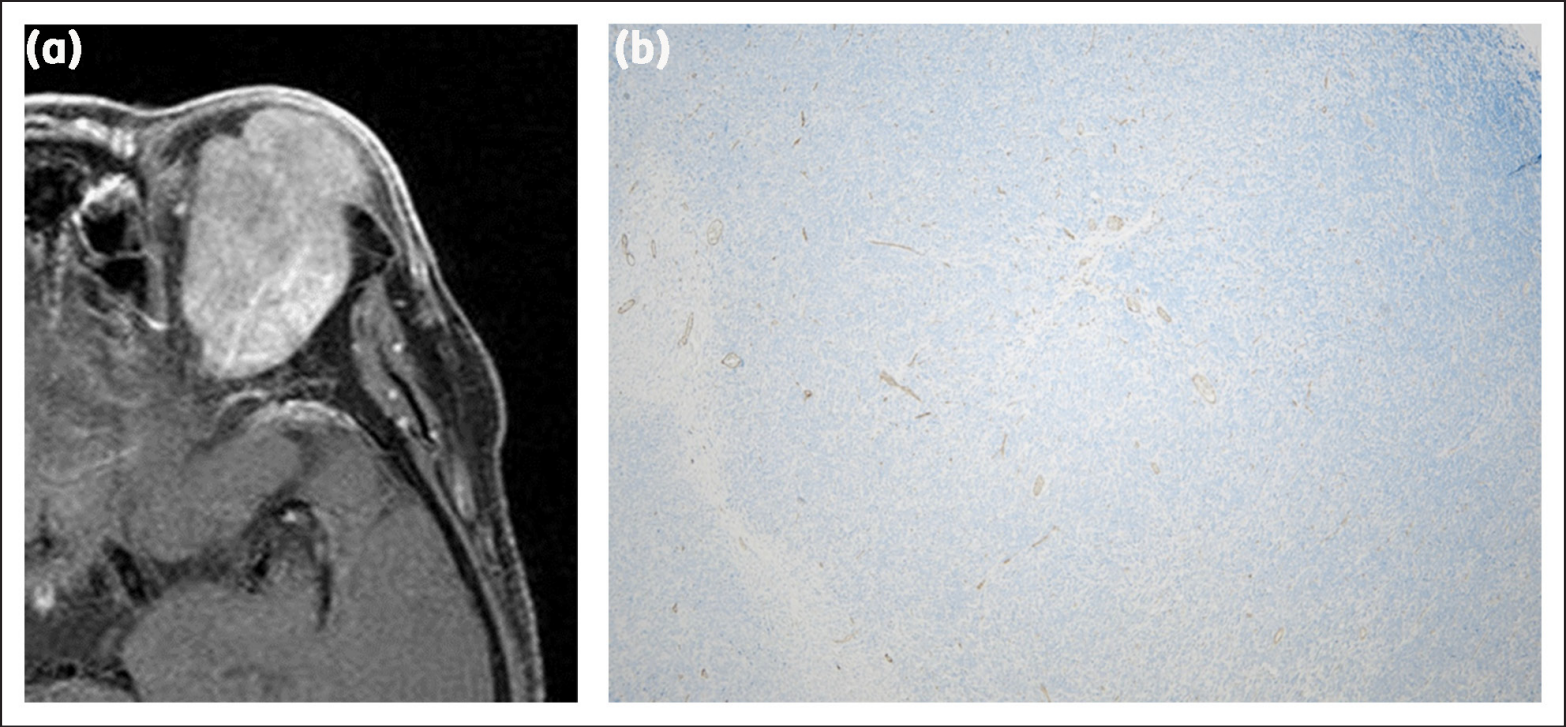
A 69-year-old woman with solitary fibrous tumor (SFT). Inhomogeneous enhancement was observed on enhanced magnetic resonance imaging (MRI) **(a)**. Although the level of CD31 staining was low **(b)**.

There was no significant correlation between the amount of collagenous tissue and signal intensity on both T1-WI (*ρ* = 0.262, *p* = 0.49) and T2-WI (*ρ* = 0.284, *p* = 0.46) (***Figures 1*** and ***2***). A significant correlation was not observed between the level of CD31 staining and homogeneity of the enhancement pattern (*ρ* = –0.13, *p* = 0.759) (***Figure 3***).

The summary of three texture analyses (histogram, co-occurrence matrix, and run-length matrix) is shown in ***Table 2***. Kurtosis in T2-WI was substantially correlated with the level of CD31 (*ρ* = –0.67, *p* = 0.048) and was strongly correlated with the amount of collagenous tissue (*ρ* = –0.97, *p* < 0.001). The other parameters were not significantly correlated with the pathological findings.

**Table 2:** Correlation between amount of collagenous tissue and the parameters of texture analysis.


		AMOUNT OF COLLAGENOUS TISSUE				P VALUE	SPEARMAN’S RANK CORRELATION COEFFICIENT

STATISTICS	PARAMETERS	1	2	3	4	*P*	*ρ*

Histogram	entropy	0.092 ± 0.006	0.116 ± 0.016	0.078 ± 0.032	0.095 ± 0.029	0.95	0.03

	skewness	0.178 ± 1.030	0.145 ± 0.597	0.109 ± 0.292	0.185 ± 0.311	0.62	–0.20

	kurtosis	4.518 ± 0.866	3.309 ± 0.297	2.619 ± 0.022	2.504 ± 0.132	<0.0001*	–0.97

	mean	120721± 18323	81868 ± 42632	119114 ± 42736	177961 ± 31071	0.181	0.4895

	median	120020 ± 15220	81090 ± 44357	119085 ± 44357	175631 ± 27137	0.181	0.4895

	0 percentile	58395 ± 37998	28688 ± 30112	61838 ± 18212	84660 ± 44717	0.548	0.2319

	25 percentiles	108099 ± 13912	70731 ± 46025	104550 ± 38947	154658 ± 29030	0.181	0.4895

	50 percentiles	120020 ± 15220	81090 ± 44357	119085 ± 44357	175631 ± 27137	0.181	0.4895

	75 percentiles	131771 ± 19874	92183 ± 41292	133652 ± 46836	200303 ± 31555	0.181	0.4895

	100 percentiles	198390 ± 44906	131708 ± 27227	180540 ± 62027	266730 ± 40029	0.2703	0.4122

Co–occurrence matrix	contrast	0.100 ± 0.009	0.149 ± 0.058	0.102 ± 0.095	0.118 ± 0.040	0.965	–0.0172

	correlation	0.951 ± 0.010	0.942 ± 0.023	0.910 ± 0.040	0.955 ± 0.001	0.4182	–0.3092

	energy	0.975 ± 0.002	0.968 ± 0.006	0.984 ± 0.017	0.974 ± 0.010	0.9475	–0.0258

	homogeneity	0.995 ± 0.000	0.993 ± 0.001	0.997 ± 0.004	0.994 ± 0.003	0.9475	–0.0258

Run-length matrix	SLE	0.409 ± 0.021	0.326 ± 0.029	0.439 ± 0.058	0.403 ± 0.092	0.7412	0.1288

	LRE	65.445 ± 13.718	75.242 ± 40.237	68.610 ± 7.421	74.700 ± 7.116	0.5636	0.2233

	GLN	274.808 ± 38.795	467.219 ± 38.782	271.958 ± 25.069	332.819 ± 192.982	0.9825	–0.0086

	RP	313.761 ± 15.303	307.426 ± 56.256	391.400 ± 99.577	307.451 ± 6.512	0.758	0.1202

	RLN	0.259 ± 0.008	0.327 ± 0.030	0.246 ± 0.047	0.275 ±0.082	0.9475	0.0258

	LGRE	0.442 ± 0.033	0.534 ± 0.001	0.395 ± 0.069	0.458 ±0.102	0.758	–0.1202

	HGRE	20213 ± 383	15724 ± 1735	38253 ± 27746	19984 ± 6676	0.9475	–0.0258

	SRLGLE	0.009 ± 0.002	0.010 ± 0.004	0.008 ± 0.005	0.011 ± 0.009	0.8433	–0.0773

	SRHGLE	20211 ± 383	15722 ± 1735	38251 ± 27746	19982 ± 6676	0.9475	–0.0258

	LRLGLE	20211 ± 383	15722 ± 1735	38251 ± 27746	19982 ± 6676	0.9475	–0.0258

	LRHGLE	20432 ± 372	15932 ± 1588	38530 ± 27752	20225 ± 6712	0.9475	–0.0258


* p < 0.05. Four categories of the proliferation of collagenous fibers or fibrous components: (1), myxoid change (2), small amount of collagenous tissue (3), moderate amount of collagenous tissue and (4), abundant collagenous tissue. SLE: Short-run emphasis, LRE: Long-run emphasis, GLN: Gray-level non uniformity, RP: Run percentage, RLN: Run length non-uniformity. LGRE: Low gray-level run emphasis, HGRE: High gray-level run emphasis. SRLGLE: Short-run low gray-level emphasis, SRHGLE: Short-run high gray-level emphasis, LRLGLE: Long-run low gray-level emphasis, LRHGLE: Long-run high gray-level emphasis.

## Discussion

The qualitative signal intensity on T2WI and the amount of collagenous tissue confirmed pathologically showed no significant correlation. However, the kurtosis in the histogram analysis showed a strong correlation with the amount of collagenous tissue. This result indicates that the texture analysis may allow the characterization of SFTs, which is difficult by visual inspection. On the other hand, high-level texture analyses such as the co-occurrence matrix and the run-length matrix did not show significant correlation with pathological findings. We supposed that this result had been reflected by the random distribution of collagenous tissue in the SFTs.

The signal intensities displayed on T1-WI were mainly isointense and some were mildly hypointense or mildly hyperintense. Previous studies reported homogeneous isointensity [4,9] or mild hypointensity [10,11] in SFTs on T1-WI. Previously reported cases did not show hyperintensity. SFTs sometimes undergo cystic or hemorrhagic changes [12], which may be seen on T1-WI. Furthermore, signal intensities were determined based on the intensity of either the cerebral cortical region or muscle, and this inconsistency may have caused variable results in previous reports.

SFTs are well-defined masses located in various areas of the retrobulbar space. Zhang et al. reported that SFTs are always located in the superior retrobulbar region [6]. Seven of the nine patients with SFTs in the present study had lesions in the superior portion of the retrobulbar space, which is consistent with previous reports. Two of the nine SFTs were located in the retrobulbar intraconal space and seven in the extraconal space. These results were consistent with previous reports showing that SFTs located in the extraconal space are more frequent than those in the intraconal space [4,9].

The homogeneity of enhancement was independent of the level of CD31 staining. SFTs’ neoplastic cells are negative for CD31 [12,13]. However, stromal vascular cells were positive for CD31 in our cases. Therefore, we assumed that vascular cells were normal vascular cells rather than neoplastic cells. Whether the number of cells positive for CD31 would affect the homogeneity of enhancement or not is still unclear.

Kurtosis in T2WI and the level of CD31 staining showed a substantial correlation. The reason of this result was the strong correlation (*ρ* = 0.75, *p* = 0.02) between the level of CD31 staining and the amount of collagenous tissue. This result might reflect the relation between neovascularization and growth of stroma.

The present study has some limitations. First, the number of subjects included in the study was small. Orbital SFTs are relatively rare and previous studies also analyzed a relatively small number of subjects. Therefore, we believe that large sample sizes are necessary. Secondly, the contrast-enhanced method varied in our cases. Dynamic studies play an important role in the differential diagnoses, as SFTs usually have a hypervascular nature.

In conclusion, orbital SFTs showed a variable intensity on T2WI. Kurtosis in the histogram analysis on T2WI had a strong correlation with the amount of collagenous tissue.

## References

[1] Klemperer P, Rabin CB. Primary neoplasms of the pleura. Arch Pathol. 1931; 11: 385–412.

[2] Yalcin CE, Tihan T. Solitary Fibrous Tumor/Hemangiopericytoma Dichotomy Revisited: A Restless Family of Neoplasms in the CNS. Adv Anat Pathol. 2016; 23(2): 104–111. DOI: 10.1097/PAP.0000000000000103 26849816

[3] Westra WH, Gerald WL, Rosai J. Solitary fibrous tumor. Consistent CD34 immunoreactivity and occurrence in the orbit. Am J Surg Pathol. 1994; 18(10): 992–998. DOI: 10.1097/00000478-199410000-00003 7522416

[4] Kim HJ, Kim HJ, Kim YD, et al. Solitary fibrous tumor of the orbit: CT and MR imaging findings. AJNR Am J Neuroradiol. 2008; 29(5): 857–862. DOI: 10.3174/ajnr.A0961 18272558 PMC8128581

[5] Bernardini FP, de Conciliis C, Schneider S, Kersten RC, Kulwin DR. Solitary fibrous tumor of the orbit: Is it rare? Report of a case series and review of the literature. Ophthalmology. 2003; 110(7): 1442–1448. DOI: 10.1016/S0161-6420(03)00459-7 12867407

[6] Zhang Z, Shi J, Guo J, Yan F, Fu L, Xian J. Value of MR imaging in differentiation between solitary fibrous tumor and schwannoma in the orbit. AJNR Am J Neuroradiol. 2013; 34(5): 1067–1071. DOI: 10.3174/ajnr.A3340 23306015 PMC7964636

[7] Baessler B, Luecke C, Lurz J, et al. Cardiac MRI and Texture Analysis of Myocardial T1 and T2 Maps in Myocarditis with Acute versus Chronic Symptoms of Heart Failure. Radiology. 2019. DOI: 10.1148/radiol.2019190101 31361205

[8] Chang JY, Kessler HP. Masson trichrome stain helps differentiate myofibroma from smooth muscle lesions in the head and neck region. J Formos Med Assoc. 2008; 107(10): 767–773. DOI: 10.1016/S0929-6646(08)60189-8 18926943

[9] Yang BT, Wang YZ, Dong JY, Wang XY, Wang ZC. MRI study of solitary fibrous tumor in the orbit. AJR Am J Roentgenol. 2012; 199(4): W506–511. DOI: 10.2214/AJR.11.8477 22997401

[10] Liu Y, Tao X, Shi H, Li K. MRI findings of solitary fibrous tumours in the head and neck region. Dentomaxillofac Radiol. 2014; 43(3). DOI: 10.1259/dmfr.20130415 PMC4064629 24720608

[11] Liu Y, Li K, Shi H, Tao X. Solitary fibrous tumours in the extracranial head and neck region: correlation of CT and MR features with pathologic findings. Radiol Med. 2014; 119(12): 910–919. DOI: 10.1007/s11547-014-0409-9 24862631

[12] Thway K, Ng W, Noujaim J, Jones RL, Fisher C. The Current Status of Solitary Fibrous Tumor: Diagnostic features, variants, and genetics. Int J Surg Pathol. 2016; 24(4): 281–292. DOI: 10.1177/1066896915627485 26811389

[13] Hanau CA, Miettinen M. Solitary fibrous tumor: Histological and immunohistochemical spectrum of benign and malignant variants presenting at different sites. Hum Pathol. 1995; 26(4): 440–449. DOI: 10.1016/0046-8177(95)90147-7 7705824

